# Supplementary material for: Biased reorientation in the chemotaxis of peritrichous bacteria Salmonella enterica serovar Typhimurium
Source: Biophys J. 2021 May 6;120(13):2623–30. doi: 10.1016/j.bpj.2021.04.033 (PMC8390861; doi:10.1016/j.bpj.2021.04.033)
Supplement: Document S1. Supporting materials and methods and Figs S1 and S2 [file mmc1.pdf]

Biophysical Journal, Volume *120*

Supplemental information

Biased reorientation in the chemotaxis of peritrichous bacteria *Salmonella enterica* serovar Typhimurium

Tonau Nakai, Taishi Ando, and Tomonobu Goto

Biased re-orientation in the chemotaxis of peritrichous bacteria *Salmonella enterica* serovar Typhimurium

Tonau Nakai, Taishi Ando, and Tomonobu Goto (Tottori University)

Supporting Materials

S1. Diffusion around the capillary tip

Figure S1 shows the snapshot of diffusion of fluorescein and the time variation of the fluorescence intensity distribution. The fluorescence distribution around the tip seems approximately concentric, which continued at least for 20 minutes. The light intensity monotonically decreases along the line OP (~ 500 μm). The diffusion constant is inversely proportional to the size of the particle. The size of L-serine is roughly estimated to be 0.7 times as large as that of fluorescein if both molecules are spherical (cubic root of the ratio of the molecular weight: ($\{105/332\}^{1/3} \approx 0.681$). Thus, similar concentration gradient of an attractant is also assumed in the observation of the chemotactic behavior of cells.

S2. Three dimensional measurement of the turn angles

For the reason that in Fig. 5 *C*, the depth of direction is not shown in the turn angle distribution (the obtained angles are different from the actual one), we performed three-dimensional measurements and verified the validity of the angle measurement by two-dimensional observation.

A piezo element (THK PRECISION, PFHW2020-400U-S) was installed between the objective lens (OLYMPUS, LUCPlanFLN40X) and revolver of the inverted microscope (OLYMPUS, IX73), and connected to the PC via the controller (THK PRECISION, NCS6111S). The objective lens was vertically oscillated (period T of 0.125 s and amplitude of 50 μm) by a sinusoidal voltage applied to the piezo while recording with a high-speed camera (Photron, FASTCAM SA-X2) at 2000 fps. Images where the cell was in focus were extracted from the obtained movie, and then the z-position of the cell was deduced. The moments of the tumbles were judged based on the swimming trajectory. The judgment based on the change in the swimming speed (see Fig.2) was not carried out because the time resolution of the three-dimensional measurement (approximately the order of T) is as coarse as the tumble duration (~ 0.1 s).

In Fig. S2, the cumulative distribution of turning angles for each swimming direction (approaching or moving away) is shown. The angle distribution “2D” calculated from the trajectory projected on the two-dimensional plane is also shown together with the angle distribution calculated from the three-dimensional trajectory “3D”. Although there is some difference in the distribution between 3D and 2D, it is considered that the angle distribution used for the random-walk simulation is not very different.

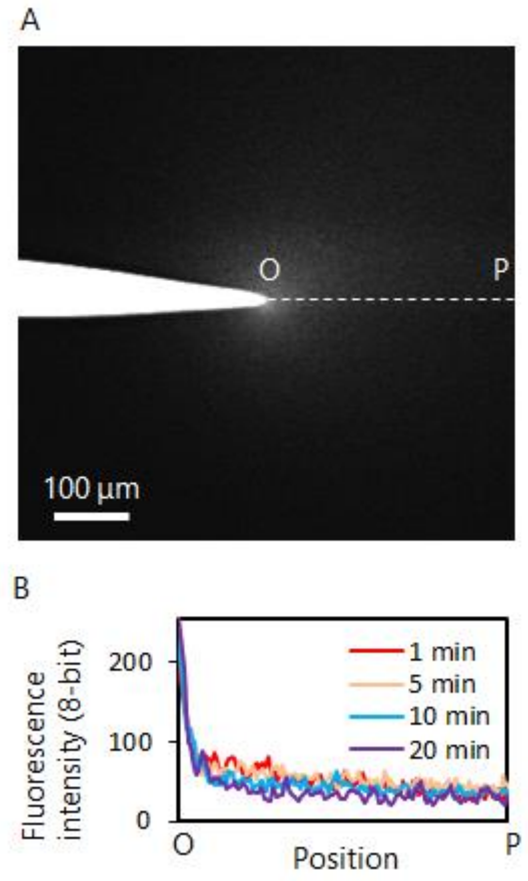

Fig. S1 Visualization of diffusion from the tip of the capillary by using fluorescein. (*A*) Fluorescent image. (*B*) Time variation of the light intensity distribution along the line OP in panel *A*.

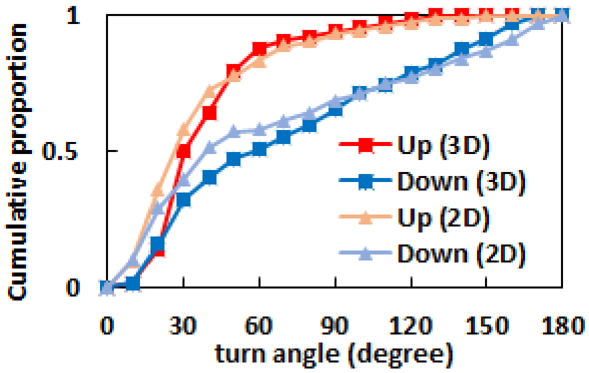

Fig. S2 Cumulative distribution of the three-dimensionally measured turn angles. “2D” means the projection of the “3D”; the turn angles were deduced by using only the two dimensional coordinates (*x* and *y*) in the 3D measurement.
